# Auditory habituation to simple tones: reduced evidence for habituation in children compared to adults

**DOI:** 10.3389/fnhum.2013.00377

**Published:** 2013-07-19

**Authors:** Jana Muenssinger, Krunoslav T. Stingl, Tamara Matuz, Gerhard Binder, Stefan Ehehalt, Hubert Preissl

**Affiliations:** ^1^fMEG Center, Institute of Medical Psychology and Behavioral Neurobiology, University of TuebingenTuebingen, Germany; ^2^Pediatric Endocrinology and Diabetes, University Children's Hospital TuebingenTuebingen, Germany; ^3^Department of Pediatrics, Dental Health Care and Social Services, Public Health Department of StuttgartStuttgart, Germany

**Keywords:** brain development, auditory processing, magnetoencephalography, brain imaging, auditory response decrement

## Abstract

Habituation—the response decrement to repetitively presented stimulation—is a basic cognitive capability and suited to investigate development and integrity of the human brain. To evaluate the developmental process of auditory habituation, the current study used magnetoencephalography (MEG) to investigate auditory habituation, dishabituation and stimulus specificity in children and adults and compared the results between age groups. Twenty-nine children (*M*_age_ = 9.69 years, SD ± 0.47) and 14 adults (*M*_age_ = 29.29 years, SD ± 3.47) participated in the study and passively listened to a habituation paradigm consisting of 100 trains of tones which were composed of five 500 Hz tones, one 750 Hz tone (dishabituator) and another two 500 Hz tones, respectively while focusing their attention on a silent movie. Adults showed the expected habituation and stimulus specificity within-trains while no response decrement was found between trains. Sensory adaptation or fatigue as a source for response decrement in adults is unlikely due to the strong reaction to the dishabituator (stimulus specificity) and strong mismatch negativity (MMN) responses. However, in children neither habituation nor dishabituation or stimulus specificity could be found within-trains, response decrement was found across trains. It can be speculated that the differences between children and adults are linked to differences in stimulus processing due to attentional processes. This study shows developmental differences in task-related brain activation and discusses the possible influence of broader concepts such as attention, which should be taken into account when comparing performance in an identical task between age groups.

## Introduction

Habituation, the “response decrement to repetitively presented stimuli” (Thompson and Spencer, 1966) is an automatic cognitive mechanism with relevance for many daily life situations. It is a basic form of learning and can give insight into general brain functioning. Therefore, neurophysiologic habituation studies (using neuroimaging techniques to evaluate the brain reaction to repeatedly presented stimulation) are often performed to investigate the development and integrity of brain functions in humans over the entire life span. Starting before birth, habituation studies were performed in fetuses in the last trimester of pregnancy, indicating that the developing brain of the fetus already shows this basic form of learning and is able to automatically distinguish between repeated and new information (Sheridan et al., 2008; Matuz et al., 2012; Muenssinger et al., 2013). During early childhood, neurophysiologic habituation studies were mainly used to address more specific questions such as early detection of impairments in language comprehension in newborns and toddlers (Benasich and Tallal, 1996). Moreover, response decrement of auditory evoked response (AER) amplitudes to repetitive stimulation could be shown in adults (Sörös et al., 2001; Rosburg et al., 2002, 2006). While fetal, neonatal and adult studies were conducted in order to answer questions concerning basic functional mechanisms of the brain; to our knowledge no study investigated the brain mechanisms of habituation in children. In this age group, the habituation process may be affected by the fact that cognitive processes as processing speed, executive functions, attention and memory (Welsh et al., 1991; Gomes et al., 2000) as well as the temporal structure of AER components (Sussman et al., 2008; Ruhnau et al., 2011) are still developing. A suitable approach to assess development-related changes in habituation is to evaluate AERs to repetitively presented stimuli using magnetoencephalography (MEG). With its high temporal resolution, this method allows a non-invasive evaluation of brain activation during habituation in both children and adults. However, while habituation is one explanation for response decrement to repetitive stimulation, also sensory adaptation or fatigue—a decrement in neuronal responsiveness due to successive stimulation—needs to be taken into account as a possible mechanism. So far, literature concerning this topic is highly inconsistent regarding the criteria used for the differentiation between these two mechanisms, which also leads to different interpretations of study results (Barry et al., 1992; Budd et al., 1998; Rosburg et al., 2010).

One criterion for habituation is, that the response decrement has to be progressive and needs to follow exponential or linear trends (Rankin et al., 2009). Moreover, two other criteria are proposed by a revision of Thompson and Spencer's (1966) criteria of habituation (Rankin et al., 2009): stimulus specificity and dishabituation. Since habituation is stimulus specific, the insertion of a deviant tone (dishabituator) into an array of repetitively presented tones should cause response recovery (criterion of “stimulus specificity”). Moreover, this insertion of a dishabituator also interrupts the habituation process, the response to a representation of the formerly habituated tone should also show response recovery compared to the last presentation of this tone before the dishabituator (criterion of “dishabituation”). If response decrement would be due to sensory adaptation/fatigue, it would continue even if a dishabituator of the same modality was inserted in the array of standard tones and also no response recovery for the standard tone after the dishabituator would occur. In contrast, if response decrement would be due to habituation, a dishabituator of the same modality but slightly different than the original stimulus (e.g., frequency differences for auditory stimulation) would elicit higher responses to this new stimulus and also the habituated stimulus after the dishabituator would experience a response recovery. Similar as “stimulus specificity”, also mismatch negativity (MMN) indicates difference detection. This pre-attentive response occurs when a sequence of standard tones is interrupted by a deviant tone and corresponds to the ability to detect differences between the two tones (Näätänen, 1992, 2001) and is generated by memory-comparison-based and N1-refractoriness effects (Schröger, 2007). Similar mechanisms like the formation of a memory trace for the frequent stimulus and the detection of change when a deviating stimulus is presented are involved in an MMN response as well as in habituation. Therefore, the occurrence of MMN responses strengthens the results found for “stimulus specificity” and can be interpreted as support for the hypothesis that stimulus decrement is due to habituation and not a result of sensory adaptation or fatigue.

Using an auditory habituation paradigm which allows for the evaluation of habituation, dishabituation, stimulus specificity and MMNs, we previously performed a study with fetuses and neonates. Results showed significant habituation and stimulus specificity already in the last trimester of pregnancy (Muenssinger et al., 2013). The current study aims to extend the evaluation of auditory habituation into child- and adulthood. Since in adolescence the highest and fastest developmental changes in the brain can be seen, heterogeneity between subjects of the same age is expected to be highest in this period. For that reason, we only included children in the pre-puberty age to decrease between-subjects heterogeneity. In order to evaluate response decrement in pre-puberty children and adults, distinguish between habituation and sensory fatigue/adaptation as cause of response decrement and gain information about development-related differences in brain processing, the same paradigm as in the previous study (Muenssinger et al., 2013) was used. Children were expected to show slower habituation (more stimuli needed before response cessation) than adults due to immature brain processes and the ongoing myelination in childhood, which may cause slower stimulus processing and delayed memory generation. The ability to differentiate between two stimuli of the same modality, and therefore to show dishabituation and stimulus specificity, was expected to be present in both groups.

## Materials and methods

### Participants

Twenty-nine healthy children between the ages of 9 and 11 years (*M*_age_ = 9.69 years, SD ± 0.47, 13 female) and 14 healthy adults between the ages of 24 and 35 years (*M*_age_ = 29.29 years, SD ± 3.47, 11 female) participated in the study. None of them took any regular medication or was diagnosed with ADHD or any other neurological disorder. Written informed consent was given before the measurement from the adult participants as well as from the children and their caregiver. The study was approved by the Ethical Committee of the Medical Faculty of the University of Tübingen.

### Data acquisition

All data was collected using a 275-sensor whole head MEG system (VSM MedTech Ltd., Port Coquitlam, Canada). Subjects were seated in a comfortable position and the head was centered in the helmet. To reduce head movement, foam pads were inserted to fixate the head. Continuous head position was recorded through localization coils attached to the nasion, left preauricular point and right preauricular point. The system was installed in a magnetically shielded room (Vakuumschmelze, Hanau, Germany) to attenuate environmental magnetic noise. Data was recorded with a sampling rate of 585 Hz.

### Procedure

Before each measurement, the device was introduced to the subjects and potential questions were answered. Before children measurements, the device as well as the camera and intercom for communication during the study were demonstrated in a child-friendly way to make the children comfortable with the new environment.

**Figure 1:**
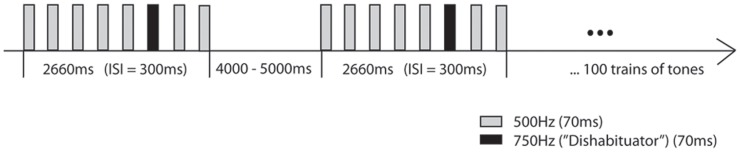
**Auditory habituation paradigm: A train of five 500 Hz tones is followed by a 750 Hz tone and two additional 500 Hz tones with an ISI of 300 ms and an ITI ranging between 4000–5000 ms (Muenssinger et al., 2013)**.

During the MEG measurement, a tone sequence consisting of 100 trains of 8 tones each was presented using non-magnetic air-based earphones. Each block of tones consisted of 5 consecutive 500 Hz tones followed by a 750 Hz tone and two more 500 Hz tones (see Figure 1).

All tones were binaurally presented with a duration of 70 ms, an inter-stimulus-interval (ISI) of 300 ms and a sound intensity of 65 dB. The interval between trains (inter-train interval, ITI) was randomized between 4000 and 5000 ms. Total stimulation duration varied between 11 and 13 min depending on the randomized ITI. The amplitudes of the M1, a magnetic AER component occurring around 100 ms after stimulus presentation, were expected to decrease between tones 1 to 5 (habituation), increase between tones 5 and 6 (stimulus specificity), and increase between tones 5 and 7 (dishabituation).

To ensure unattended perception of the auditory stimuli, participants were instructed to focus their attention on a silent movie displayed on a screen approximately 1 m in front of them.

### Data analysis

All datasets exceeding maximal head movement of 2 cm were excluded from further analysis. The data was filtered offline between 1 and 40 Hz and separated into trials starting with a baseline of 100 ms before and ending 3000 ms after presentation of the first stimulus per train. All trials with amplitudes higher than 2 pT were automatically marked as bad and excluded from further analysis. For the remaining datasets (children: 22 datasets, adults: 14 datasets for within-train analysis, 12 datasets for between-train analysis), an average of all trials per subject was created and the grand average was calculated.

All data was baseline corrected to the pre-stimulus intervals. To control for the differences in head-sensor distance between children and adults, data were normalized to the first tone per subject. All statistical analysis is based on this normalized data.

#### Within-train analysis

For statistical within-train analysis, differences between M1 amplitudes of the different tones were evaluated. Therefore, the peak with the highest amplitude within an interval between 80 and 140 ms after tone presentation was selected and root mean square (RMS) values were calculated. A 2 by 8 factor repeated-measure ANOVA with “tones” (the RMS values of the M1 of the 8 tones) as within-subjects factor and “group” (children vs. adults) as between-subjects factor was used to evaluate the influence of the different tones on M1 as well as the difference between the groups. *Post-hoc* comparisons between tone pairs to evaluate habituation (decrement between tones 1 and 5), stimulus specificity (increment between tones 5 and 6) and dishabituation (increment between tones 5 and 7) were performed using a paired *t*-test. The significance level was corrected for multiple comparisons to 0.0167 (Bonferroni correction). If a significant response decrement between tones 1 and 5 was found, linear and quadratic trends were examined to test if this response decrement was consistent with habituation (Barry et al., 1992; Rankin et al., 2009).

#### Between-train analysis

For between-train analysis, the trains of tones were divided into three blocks, each containing 30 trains. Only datasets with at least 90 trains after artifact rejection were included in further between-block analysis. Block 1 consisted of trains 1 to 30, block 2 contained trains 31 to 60 and block 3 contained trains 61 to 90. To compare between blocks, the RMS values over the whole blocks were extracted. Repeated-measure ANOVA was used to test for a main effect of blocks. Moreover, a paired *t*-test was employed for comparison of block pairs. The significance level was corrected for multiple comparisons to 0.025 (Bonferroni correction).

#### MMN analysis

To calculate MMNs, tone 5 (500 Hz tone directly before the dishabituator) was regarded as “standard before deviant”. Therefore, the AER (normalized to the interval 0–50 ms) elicited by tone 5 were subtracted from those of tone 6 (dishabituator, 750 Hz), which was regarded as “deviant”. Only peaks with latencies in the range of ±10 ms around the mean latency of the MMN component per group were regarded as valid individual MMN components and the RMS value was calculated.

## Results

Seven children had to be excluded from all further analysis because their head movement exceeded the threshold of 2 cm. Moreover, two adult datasets had to be excluded from the between-trains analysis because the required number of 90 trains after artifact rejection was not reached. Therefore further analysis was performed on the remaining 22 children (*M*_age_ = 9.68 years, SD ± 0.48) for within-train analysis and between-trains analysis, all 14 adults (*M*_age_ = 29.29 years, SD ± 3.47) for the within-train analysis and 12 adults (*M*_age_ = 29.17 years, SD ± 3.49) for between-trains analysis.

### Within-train analysis

The repeated-measures ANOVA revealed a significant main effect of “tones” on the RMS of M1 amplitudes [*F*_(4.8)_ = 19.60, *p* < 0.001]. Moreover, a significant effect of “group” [*F*_(1)_ = 22.83, *p* < 0.001] and a significant interaction between “tones” and “group” [*F*_(4.8)_ = 10.26, *p* < 0.001] have been found.

**Figure 2:**
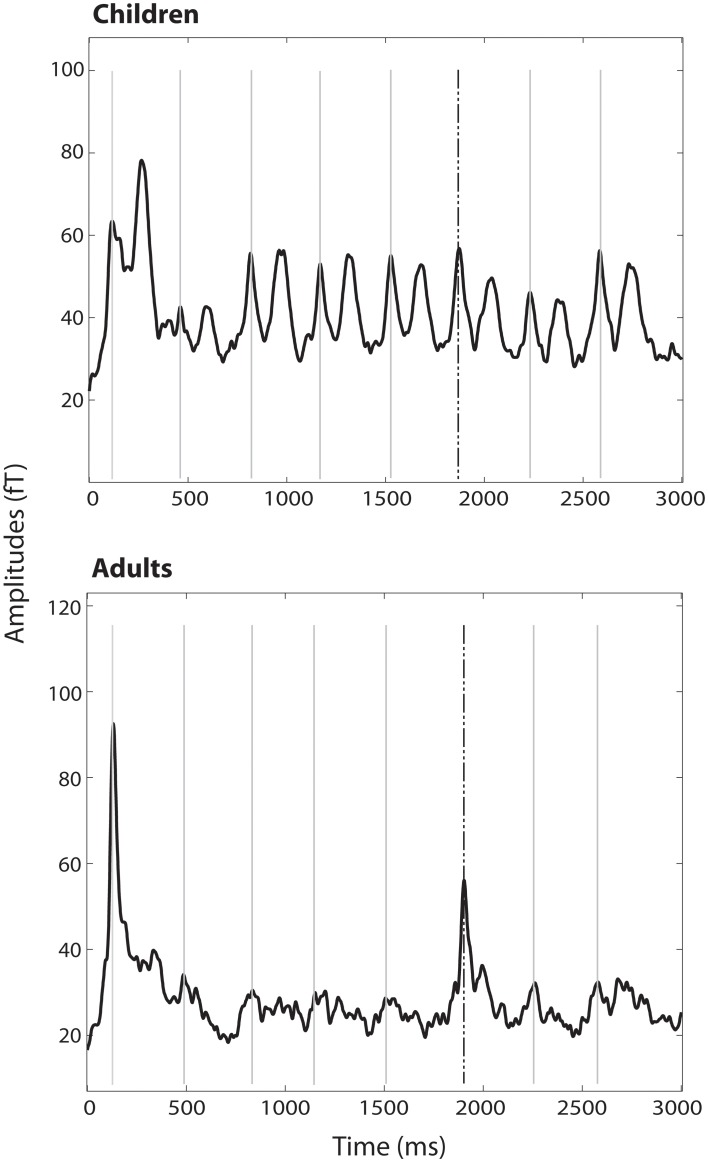
**RMS of all channels averaged over all trains and subjects per group**. M1 to the 500 Hz tones are marked with gray lines; the response to the dishabituator (750 Hz tone) is indicated by the dashed black line.

**Figure 3:**
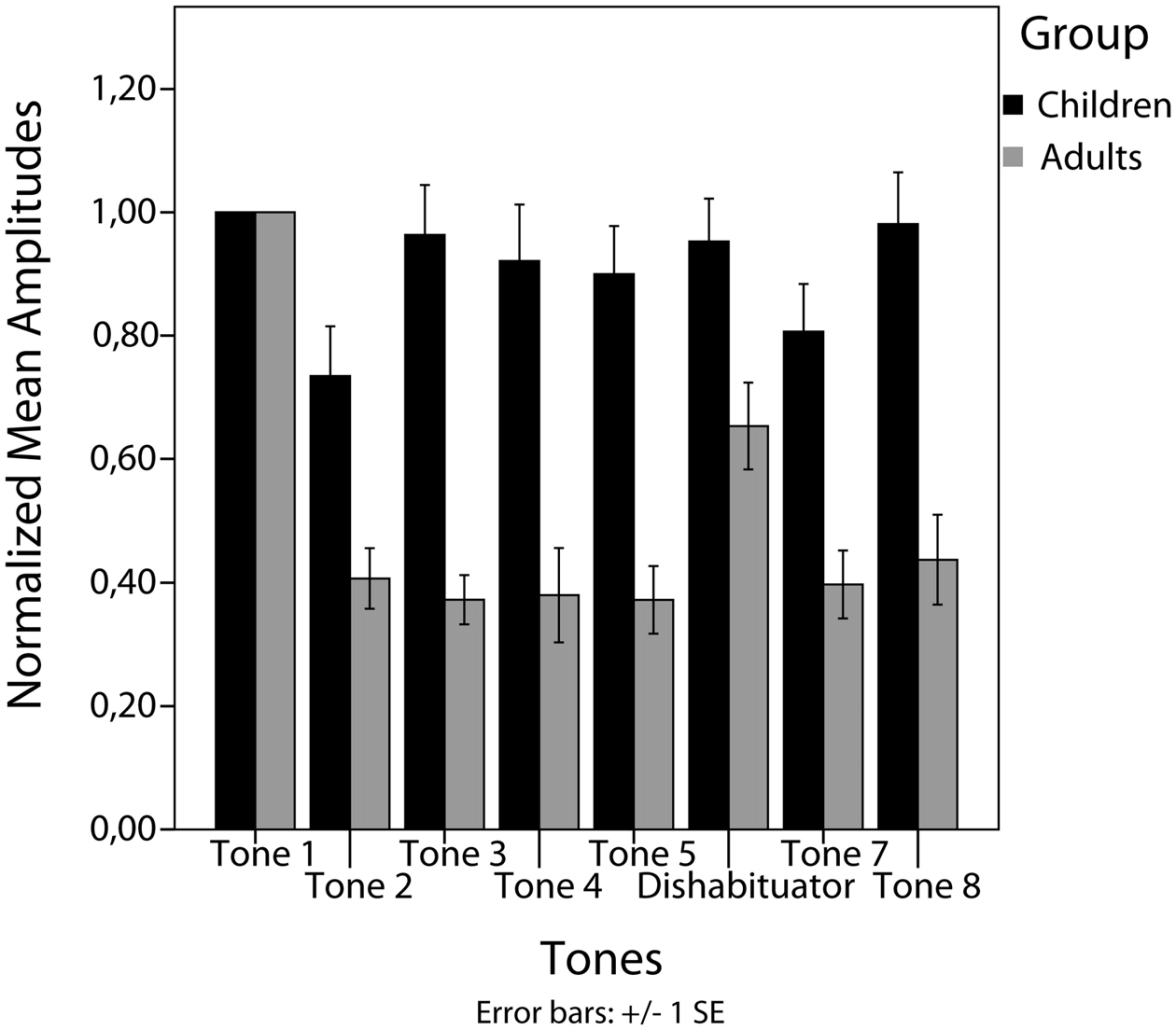
**Mean amplitudes normalized to the 1st tone per subject of the two groups**.

In the group of children no significant decrease between tone 1 and tone 5 (habituation) [*t*_(21)_ = 0.83, *p* = 0.41], no significant increase from tone 5 to tone 6 (stimulus specificity) [*t*_(21)_ = −0.35, *p* = 0.73] and no significant increase from tone 5 to tone 7 (dishabituation) [*t*_(21)_ = −1.67, *p* = 0.109] have been found (Figures 2, 3).

In contrast, in the adult group a significant decrease from tone 1 to tone 5 (habituation) [*t*_(13)_ = 14.71, *p* < 0.001] and a significant increase from tone 5 to tone 6 (stimulus specificity) [*t*_(13)_ = −4.98, *p* < 0.001] have been found. No significance was reached for the increase between tone 5 and tone 7 (dishabituation) [*t*_(13)_ = −1.25, *p* = 0.232] (Figures 2, 3). Significant linear [*F*_(1)_ = 220.22, *p* < 0.001] and quadratic [*F*_(1)_ = 171.41, *p* < 0.001] trends were found for the response decrement between tones 1 and 5.

### MMN analysis

**Figure 4:**
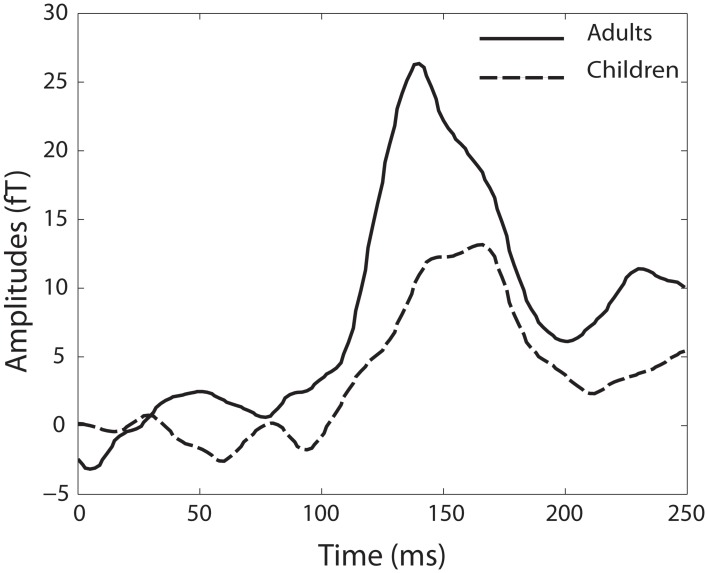
**Baseline-corrected MMN responses for the adult group (black line) and the group of children (dotted black line)**.

In the group of children, MMN responses within a range of ± 10 ms around the mean latency of 167 ms were detected in 11 of the 22 children. The mean amplitude over all children was 14.49 fT (SD ± 19.67). In the adult group, 12 of the 14 adults showed MMN responses within a range ±10 ms around the mean latency of 140 ms. The mean amplitude of all adult MMN responses was 26.34 fT (SD ± 13.39) (Figure 4).

### Between-train analysis

**Figure 5:**
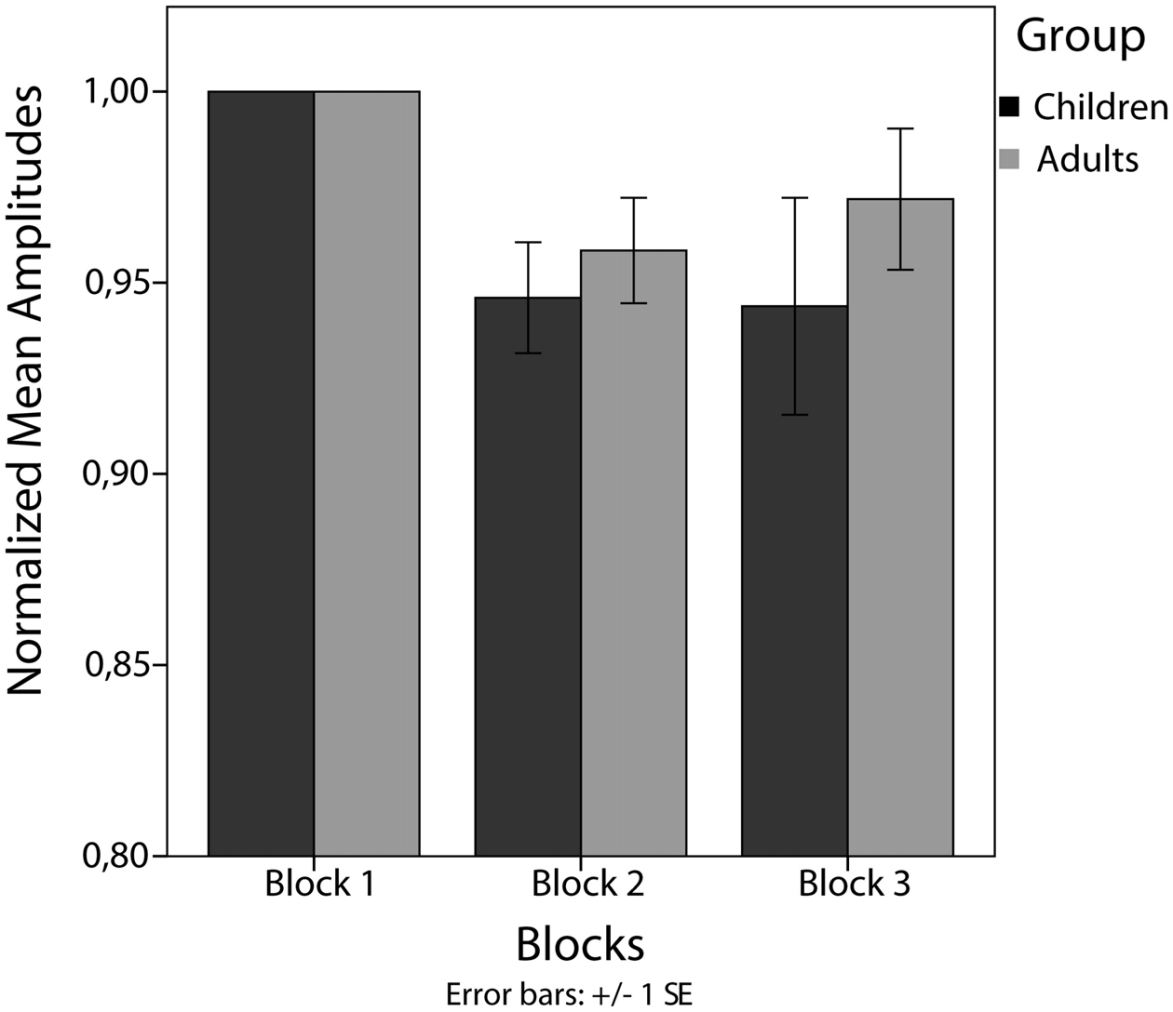
**Mean amplitudes normalized to the first block per subject**.

Between-trains analysis revealed a significant main effect of blocks in children [*F*_(2)_ = 4.45, *p* < 0.05]. Amplitudes decreased significantly from block 1 to block 2 [*t*_(20)_ = 3.71, *p* < 0.025] but not from block 2 to block 3 [*t*_(20)_ = 0.12, *p* = 0.906]. For adults, no significant main effect of blocks has been found [*F*_(2)_ = 3.04, *p* = 0.069] (see Figure 5).

## Discussion

The aim of the current study was to evaluate response decrement to repeatedly presented auditory stimulation in children, differentiate between habituation and sensory adaptation/fatigue as reason for response decrement and compare the results with adult data to gain new information about development-related differences. Children were expected to habituate slower than adults and both groups were expected to show stimulus specificity and dishabituation as a clear indicator for habituation as source of M1 amplitude decrement.

Within trains, a distinct and high reaction to the first tone per train has been found in the children as well as in the adult group. It is noteworthy though, that the course of M1 amplitudes within the trains differed between the two groups. In the group of children, no significant M1 decrement between tones 1 and 5 and also no M1 increment between the last habituated response and the dishabituator (stimulus specificity) could be found. In contrast, adults showed the expected decrement between tones 1 and 5 and the increment in M1 amplitude between tones 5 and 6 (stimulus specificity). A significant increment between tones 5 and 7 (dishabituation) could not be found in any group.

Myelination-related differences in the speed of signal conduction between children and adults cannot explain the results since this would have caused differences in the speed of response decrement but not in differences in the within-train course of M1 amplitudes between groups.

However, developmental aspects of higher cognitive functions as attentional mechanisms could be a further possible explanation for the difference between the two groups. According to the embedded-processes model (Cowan, 1999), attention is controlled by two different processes: an automatic process which is activated by novel stimuli (e.g., novel sounds) and a voluntary process which is driven by top–down control. While the automatic process is rather basic and develops early in childhood, voluntary directing ones attention to relevant stimuli and ignore irrelevant stimuli (selective attention) is a more complex process, which is still developing during childhood. A study evaluating selective attention using an auditory selection task with children between 8 and 14 years of age showed, that, even while distraction retention remained constant over the age groups, the influence of the distractor on the primary task decreased with age (Doyle, 1973). Moreover, weaker distraction control on the ERP level in children compared to adults was found even while some degree of behavioral distraction control was seen already in the group of children (Wetzel et al., 2009). This seems to be a general developmental effect [for a review see Gomes et al. (2000)]. In addition, executive “top–down” functions which are needed for selective attention are related to the prefrontal cortex (PFC), a brain region which is known to develop until late adolescence (Diamond, 2002; Gogtay et al., 2004; SanMiguel et al., 2008). This developmental change in the control of selective attention could also have influenced the results of the current study. To enable an unattended processing of the auditory stimulation, all participants were asked to concentrate on a silent movie (visual stimulation) and ignore the tones (auditory stimulation). Therefore, they were simultaneously confronted with two types of stimulation, which can be seen as a basic form of a selection task with the movie as relevant task and the auditory stimulation as distractor, which should be ignored. Because the silent movie was started shortly before the auditory stimulation in all subjects, it can be hypothesized that, due to the orienting response, all subjects' attention was shifted to the auditory stimulation when the first tone was presented (Bell et al., 2012). However, adults are known to be able to use top–down mechanisms to direct their attention back to the relevant target (in this case the movie), enabling them to watch the movie and perceive the auditory stimulation unattendedly as supposed. Since children are known to perform worse at selective attention tasks and to be more prone of attending to both, the relevant and the irrelevant stimulus streams (Gomes et al., 2000), the influence of the “to be ignored” auditory stimulation might have been higher than in the adult group. Therefore, it might be speculated, that children at least partly attended to the auditory stimulation, which may have changed stimulus processing: even though all tones were perceived (indicated by the AER response to every tone), when actively attended, the tones of one train may have been grouped and processed as one entity and not as a composition of different tones. In this case, no habituation within the trains of tones but habituation over trains would be expected. Indeed, our between-trains analysis of habituation showed this for the children. In adults we observed a significant habituation within trains but not between trains, the findings in the group of children were exactly the opposite. Moreover, the fact that M1 amplitudes from tones 3 to 8 were similar, showing no response increment for the dishabituator as well as the lack of MMN responses to the dishabituator in half of the children might be interpreted as an indicator that the tones within a train were not processed as individual stimuli by all children which in turn might indicate that the group of children was not able to focus attention on the movie to enable unattended processing of the auditory stimulation. However, to thoroughly understand the underlying brain mechanisms involved in the current results, source localization is needed to identify involved brain structures. Additionally, due to the observed trend toward significance in the adults group and its small sample size, the between-train results have to be taken with care and need to be reproduced in a bigger sample for clear interpretation. Moreover, in future studies children's ability to focus attention and their state of arousal has to be investigated and instead of presenting a movie, a visual task involving working memory could be applied since a high working memory load in the primary task was found to decrease distraction caused by irrelevant sounds (SanMiguel et al., 2008).

Another possible explanation for the missing response decrement of the M1 component in children might be that the M1 component is still in its development and does not behave maturely at an age of 10 years. However, current literature is inconsistent on the development of the M1 component (Rojas et al., 1998; Sussman et al., 2008; Rankin et al., 2009) and further research is needed to thoroughly resolve this question.

Even while children did not show habituation within trains, the expected response decrement from tone 1 to tone 5 was found in the group of adults. To examine the question if this was due to habituation or sensory adaptation/fatigue, we added a dishabituator to the paradigm, which enabled us to assess stimulus specificity, dishabituation and MMN responses. While originally the criteria of dishabituation was used in most studies to differentiate between habituation and sensory adaptation/fatigue (Thompson and Spencer, 1966), the revised criteria of habituation (Rankin et al., 2009) emphasize stimulus specificity as strong indicator for habituation. Moreover, also MMN responses can be interpreted as an evidence for habituation since an MMN response has similar requirements as habituation: the subject's brain has to be able to remember the stimulus, differentiate between different stimuli and react to the change in stimulation. In the group of adults, a significant increase in M1 amplitudes for the dishabituator (stimulus specificity) could be shown. Moreover, 12 out of the 14 adults showed a MMN response between the dishabituator and the standard before the dishabituator. However, surprisingly, in both groups no significant dishabituation was found. This might be due to the choice of the dishabituator in the current paradigm, which was chosen from the same sensory modality as the standard tone and differed only in frequency. The reason to choose this dishabituator was that similarity to the standard tones ensures that the groups of neurons reacting to the two stimuli are highly overlapping. This enables a clear statement concerning stimulus specificity and MMN responses: if response decrement would have been due to sensory adaptation/fatigue, no response decrement (and also no MMN response) would have been expected between the dishabituator and the last standard before the dishabituator since mostly the same neurons were activated by the two stimuli. However, the clear increase in response amplitude and the MMN responses found between the two stimuli shows that the brain was able to remember the standard tones and differentiate between the standard and the dishabituator. However, while the dishabituator was perfectly suitable to show stimulus specificity and elicit MMN responses, it remains still unclear in the literature if the dishabituator has to be stronger and more deviating from the standard to elicit dishabituation (Rankin et al., 2009). Therefore, the criteria of dishabituation should be reassessed in a future study using a dishabituator, which more strongly deviates from the standard tones.

To date, in the literature different characteristics are chosen to distinguish between habituation and sensory adaptation/fatigue and similar results are inconsistently discussed and interpreted (Barry et al., 1992; Budd et al., 1998; Rosburg et al., 2010). In the current study, three criteria for the distinction between habituation and sensory adaptation/fatigue were chosen according to the revised version of Thompson and Spencer's (1966) criteria of habituation (Rankin et al., 2009). In this revision, special emphasis is laid on “stimulus specificity” as “critical aspect of habituation”. Additionally, MMN responses were assessed as an extra indicator for habituation in the current study. Since in the adult group significant linear and quadratic trends were found for response decrement between tones 1 and 5, significant stimulus specificity was shown and MMN responses between the last habituated tone before the dishabituator and the dishabituator itself have been shown, we interpret these findings as a strong indicator for habituation as cause of response decrement.

In summary, the current study shows strong differences in the auditory habituation process between children and adults. Due to possible differences in the ability to focus attention between the groups, adult participants may have processed the stimuli as trains of individual tones, while children may have processed each train as one entity. This might indicate that not only mechanisms directly connected with stimulus processing but also broader concepts as attentional differences between adults and children should be taken into account whenever their performances are compared.

### Conflict of interest statement

The authors declare that the research was conducted in the absence of any commercial or financial relationships that could be construed as a potential conflict of interest.
